# ZFAS1 functions as an oncogenic long non-coding RNA in bladder cancer

**DOI:** 10.1042/BSR20180475

**Published:** 2018-05-15

**Authors:** Haifan Yang, Ge Li, Bo Cheng, Rui Jiang

**Affiliations:** Department of Urology, The Affiliated Hospital of Southwest Medical University, Luzhou 646000, Sichuan, China

**Keywords:** bladder cancer, biomarker, lncRNA, ZFAS1

## Abstract

Long non-coding RNA (lncRNA) ZFAS1 (zinc finger antisense 1) has been suggested to have an oncogenic role in the tumorigenesis of human malignant tumors. However, the expression status and biological function of ZFAS1 in bladder cancer is still unknown. Thus, the purpose of the present study is to explore the clinical value of ZFAS1 in bladder cancer patients, and the biological function of ZFAS1 in bladder cancer cell. In the present study, we found ZFAS1 expression was increased in bladder cancer tissues compared with paired adjacent normal tissues through analyzing the Cancer Genome Atlas (TCGA) database. Furthermore, we confirmed that levels of ZFAS1 expression were elevated in bladder cancer tissues and cell lines compared with normal bladder tissues and normal uroepithelium cell line, respectively. Then, we observed that the expression level of ZFAS1 was positively associated with clinical stag, muscularis invasion, lymph node metastasis, and distant metastasis in bladder cancer patients. The experiments *in vitro* suggested that knockdown of ZFAS1 repressed bladder cancer cell proliferation via up-regulating KLF2 and NKD2 expression, and inhibited cell migration and invasion via down-regulating ZEB1 and ZEB2 expression. In conclusion, ZFAS1 is overexpressed in bladder cancer, and functions as an oncogenic lncRNA in regulating bladder cancer cell proliferation, migration, and invasion.

## Introduction

Bladder cancer is much more common in men than in women [1]. In men, bladder cancer is the third most common cancer and the seventh leading cause of cancer-related deaths in developed countries [2,3]. Based on cancer statistics in China, there were an estimated 80500 new bladder cancer cases and 32900 deaths in 2015 [4]. Moreover, an increasing incidence trend for bladder cancer was observed from 2000 to 2011, but the age-standardized mortality rate of bladder cancer was stable [4]. Bladder cancer is the commonest amongst urinary system malignant tumors in our country [5]. Surgery combined with chemotherapy still is the major treatment strategy for bladder cancer patients, but the long-term survival is not as good as expected for most bladder cases [6–8]. Thus, it is necessary to elucidate the molecular mechanisms of bladder cancer carcinogenesis for developing target treatment.

Long non-coding RNAs (lncRNAs) are non-protein coding RNAs with more than 200 nts in length, but bear the ability to up/down-regulate gene expression [9,10]. LncRNA zinc finger antisense 1 (ZFAS1) is a transcript antisense to the 5′-end of the gene zinc finger NFX1-type containing 1 (ZNFX1). Originally, ZFAS1 was first identified by Askarian-Amiri et al. (in 2011) [11], at that time, it was suggested to be lowly expressed in breast cancer, and functioned as an antitumor effect on cell proliferation and differentiation. Recently, more and more studies conformably reported that ZFAS1 was overexpressed in most types of human cancers, and promoted tumor cell growth and metastasis [12,13]. However, the expression status and biological function of ZFAS1 in bladder cancer remain unclear. In the early experiment, we explored ZFAS1 expression in bladder cancer tissues through analyzing the Cancer Genome Atlas (TCGA) database, and found ZFAS1 expression was increased in bladder cancer tissues compared with paired adjacent normal tissues. We supposed that ZFAS1 may serve as an oncogenic lncRNA in bladder cancer. The aim of our study is to analyze the association between lncRNA ZFAS1 expression and clinicopathological characteristics in bladder cancer patients, and explored the biological role in regulating bladder cancer cell proliferation, migration, and invasion.

## Materials and methods

### TCGA database analysis

The lncRNA microarray of human hepatocellular carcinoma was obtained from TCGA projects (https://cancergenome.nih.gov/). It contained 19 pairs of hepatocellular carcinoma tissues and adjacent normal hepatic tissues. Aberrantly expressed lncRNAs were screened and identified.

### Clinical sample collection

Fresh clinical samples of 20 normal bladder tissue samples and 102 bladder cancer samples were obtained from the Affiliated Hospital of Southwest Medical University, Luzhou City, China. All specimens had confirmed pathological diagnosis and were staged according to the AJCC Cancer Staging Manual (7th edition). No bladder cancer patient underwent antitumor therapy before diagnosis. The clinical processes were approved by the Ethics Committee of the Affiliated Hospital of Southwest Medical University. Patients provided informed, written consents.

### Cell culture

The human bladder cancer cell lines (T24, RT4, J82, and SW780) were purchased from the Chinese Academy of Sciences, and maintained in RPMI 1640 medium containing 10% FBS (ExCell, China). The human normal uroepithelium cell line (SV-HUC-1) was purchased from American Type Culture Collection, maintained in F-12K medium (Gibco, U.S.A.) supplemented with 10% FBS (ExCell, China). All cell lines were cultured at 37°C in a humidified atmosphere of 5% CO_2_.

### RNA isolation and qRT-PCR

RNA was extracted from tissues and cells using TRIzol (Takara, Japan) per the manufacturer’s protocol. The cDNA was synthesized from 1 μg total RNA with PrimeScript® RT Reagent Kit (Takara, Japan). Real-time PCR was performed with the SYBR® Premix Ex Taq™ II (Takara, Japan) detection method on an ABI-7500 RT-PCR system. *GAPDH* gene was used as gene internal control. All the primers were listed as follows: ZFAS1 forward, 5′-ACGTGCAGACATCTACAACCT-3′, reverse, 5′-TACTTCCAACACCCGCAT-3′; GAPDH forward, 5′-GGAGCGAGATCCCTCCAAAAT-3′, reverse, 5′-GGCTGTTGTCATACTTCTCATGG-3′.

### Transient transfection

siRNA for ZFAS1 was designed and synthesized by Guangzhou RiboBio Inc, China. Three siRNAs targetting *ZFAS1* gene were designed and synthesized, the most effective siRNA (si-ZFAS1) identified by real time-PCR was applied for further experiments. Transfections were carried out using Lipofectamine 3000 (Invitrogen, U.S.A.) based on the manufacturer’s protocol. After 48–72 h, cells were harvested for the subsequent experiments.

### Cell proliferation analysis

MTT assay was used to evaluate cell proliferation *in vitro*. Cells were seeded in 96-well plates at a density of 1000 cells/well. Transfected cells were incubated for 4 days. Twenty microliters MTT (5 mg/ml, Sigma, U.S.A.) was added to each well and incubated for 4 h. At the end of incubation, supernatants were removed, and 150 μl DMSO (Sigma, U.S.A.) was added to each well. The absorbance value (OD) of each well was measured at 490 nm. Experiments were performed thrice.

### Cell cycle assay

To evaluate cell cycle distributions, a total number of 5 × 10^6^ cells were harvested after transfection for 48 h. Transfected cells were fixed in 70% ice-cold ethanol for 48 h at 4°C and stained with PBS containing 10 μg/ml propidium iodide and 0.5 mg/ml RNase A for 15 min at 37°C. FACS caliber flow cytometry (BD Biosciences, U.S.A.) was used to gain the DNA content of labeled cells. Each experiment was done in triplicate.

### Cell migration and invasion assays

For cell migration assays, 1 × 10^5^ transfected cells suspended in 100 μl RPMI 1640 medium without FBS were plated in a fibronectin-coated polycarbonate membrane insert in a transwell apparatus (CoStar, U.S.A.), and medium supplemented with 10% FBS was placed into the lower chamber. After the cells were incubated for 12 h at 37°C in a 5% CO_2_ atmosphere, lower surfaces of the insert were washed with PBS, fixed with methanol, stained with Giemsa solution, and counted under a microscope in five predetermined fields. For the cell invasion assay, the procedure was similar to the cell migration assay, except that the membranes of transwell apparatus were precoated with 24 μg/μl Matrigel. All assays were independently repeated three times.

### Western blot

The culture medium of cells was discarded 48 h after transfection. Transfected cells were lysed, and total proteins were extracted using RIPA protein extraction reagent (Beyotime, China) and were quantitated using the BCA protein assay kit (Beyotime, China). Equal amounts (50 μg) of protein extracts were separated by SDS/PAGE (10% gel) and transferred on to PVDF membrane. The PVDF membranes were incubated for 12 h in blocking solution, and then probed with the following primary antibodies: KLF2, NKD2, ZEB1, ZEB2, E-cadherin, Vimentin, and β-actin antibodies (1:500, Cell Signaling Technology, U.S.A.) at 4°C overnight. An HRP-conjugated IgG antibody was used as the secondary antibody (ZSGB-bio, China). Signals were detected using ECL reagents (Pierce, U.S.A.).

### Statistical analysis

Statistical analyses were performed using SPSS 17.0 software. The Wilcoxon signed rank test was performed to compare the expression of ZFAS1 between tumor tissue samples and paired adjacent normal tissue samples. The relationship between ZFAS1 and clinicopathological characteristics was analyzed using Chi-square test. Two-tailed Student’s *t* test was used for comparisons of two independent groups. A *P*-value of less than 0.05 was considered statistically significant.

## Results

### The status of ZFAS1 expression in bladder cancer tissues and cell lines

**Figure 1:**
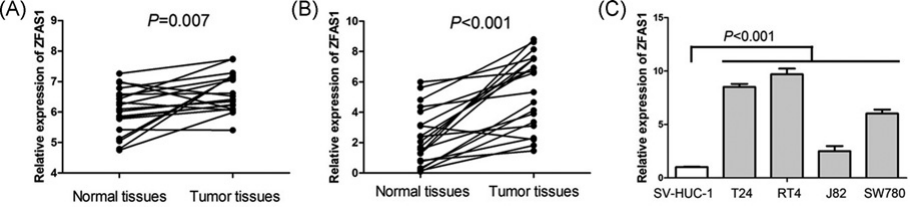
The status of ZFAS1 expression in bladder cancer tissues and cell lines (**A**) TCGA database suggests ZFAS1 expression is increased in bladder cancer tissues compared with paired adjacent normal tissues (*n*=19). (**B**) Levels of ZFAS1 expression are elevated in bladder cancer tissue samples compared with paired normal bladder tissue samples (*n*=20). (**C**) Bladder cancer cell lines suggest higher expression levels of ZFAS1 compared with normal uroepithelium cell line.

In order to identify reliable biomarkers for bladder cancer, we analyzed TCGA database, and found ZFAS1 expression was increased in bladder cancer tissues compared with paired adjacent normal tissues (*P*=0.007, Figure 1A). Furthermore, we confirmed the expression of ZFAS1 in 20 pairs of bladder cancer tissue samples and normal bladder tissue samples, and found that levels of ZFAS1 expression were elevated in bladder cancer tissue samples (*P*<0.001, Figure 1B). Moreover, we also performed qRT-PCR to measure the expression of ZFAS1 in human bladder cancer cell lines (T24, RT4, J82, and SW780) and human normal uroepithelium cell line (SV-HUC-1), and observed that bladder cancer cell lines suggested higher expression levels of ZFAS1 compared with normal uroepithelium cell line (*P*<0.001, Figure 1C).

### The relationship between ZFAS1 expression and clinicopathologic characteristics in bladder cancer

To further explore the clinical significance of ZFAS1 expression in bladder cancer, we divided all the patients into two groups according to ZFAS1 expression: the ZFAS1 high expression group (*n*=51) and the ZFAS1 low expression group (*n*=51) according to a published study [14]. The correlation between ZFAS1 expression and clinical parameters was analyzed and summarized in Table 1. There was no significant correlation between ZFAS1 expression with patient’s gender, age, tumor size, histological grade, and multiplicity in 102 bladder cancer cases. However, we observed that the expression level of ZFAS1 was positively associated with clinical stage (*P*<0.001), muscularis invasion (*P*<0.001), lymph node metastasis (*P*<0.001), and distant metastasis (*P*=0.007) in bladder cancer patients.

**Table 1:** Association between lncRNA ZFAS1 expression and clinicopathological characteristics in bladder cancer patients

Characteristics	*n*	ZFAS1 expression		*P*
		High (%)	Low (%)	
Gender				
Female	35	14 (40.0)	21 (60.0)	0.144
Male	67	37 (55.2)	30 (44.8)	
Age (years)				
<50	48	21 (43.8)	27 (56.3)	0.234
≥50	54	30 (55.6)	24 (44.4)	
Clinical stage				
I–II	46	13 (28.3)	33 (71.7)	<0.001
III–IV	56	38 (67.9)	18 (32.1)	
Tumor size				
<3 cm	53	26 (49.1)	27 (50.9)	0.843
≥3 cm	49	25 (51.0)	24 (49.0)	
Muscularis invasion				
Negative	39	9 (23.1)	30 (76.9)	<0.001
Positive	63	42 (66.7)	21 (33.3)	
Lymph node metastasis				
Negative	88	37 (42.0)	51 (58.0)	<0.001
Positive	14	14 (100)	0 (0)	
Distant metastasis				
Absent	93	42 (45.2)	51 (54.8)	0.005
Present		9	9 (100)	0 (0)
Histological grade				
High grade	68	30 (44.1)	38 (55.9)	0.093
Low grade	34	21 (61.8)	13 (38.2)	
Mutiplicity				
Single	77	39 (50.6)	38 (49.4)	0.818
Multiple	25	12 (48.0)	13 (52.0)	

### The biological function of ZFAS1 in bladder cancer cell

**Figure 2:**
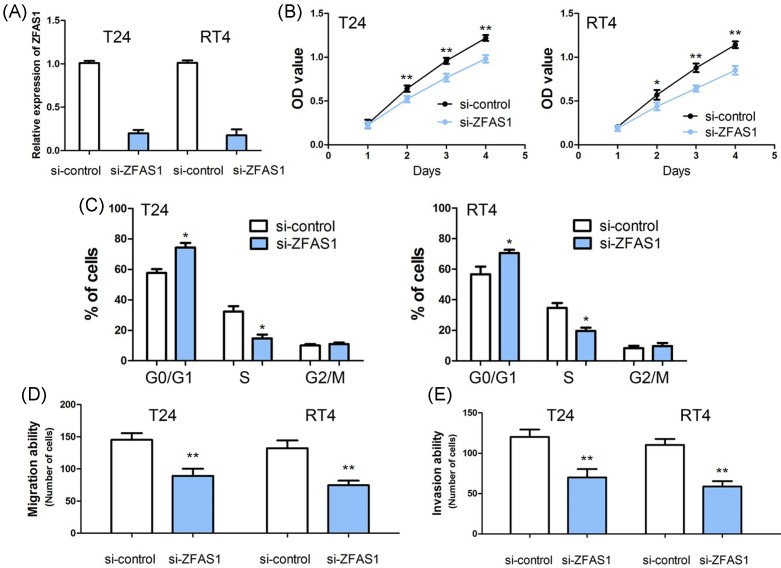
The biological function of ZFAS1 in bladder cancer cell (**A**) The efficiency of siRNA-ZFAS1 is confirmed by qRT-PCR in T24 and RT4 cells. (**B**) ZFAS1 suppression obviously decreases cell proliferation compared with the control groups. (**C**) Knocking down ZFAS1 dramatically reduces cell cycle progression from G_1_ to S phases. (**D**) Knocking down ZFAS1 markedly inhibits migratory ability in T24 and RT4 cells. (**E**) Knocking down ZFAS1 strikingly depresses invasive ability in T24 and RT4 cells. (*, *P*<0.05; **, *P*<0.001)

According to the expression of ZFAS1 in bladder cancer cell lines (T24, RT4, J82, and SW780), we found that ZFAS1 expression was relatively increased in T24 and RT4 cells. Thus, T24 and RT4 cells were used for loss-of-function studies. To test the biological functions of ZFAS1 *in vitro*, we induced down-regulation of ZFAS1 in T24 and RT4 cells through siRNA. Quantitative real-time PCR was performed to confirm the transfection efficiency of si-ZFAS1 in these lines, and the level of ZFAS1 expression was significantly decreased in si-ZFAS1 T24 and RT4 cells (Figure 2A).

We examined the effect of down-regulation of ZFAS1 on T24 and RT4 cells proliferation. Using an MTT assay, we found that ZFAS1 suppression obviously decreased cell proliferation compared with the control groups (*P*<0.05, Figure 2B). Cell cycle analysis suggested that knocking down ZFAS1 dramatically reduced cell cycle progression from G_1_ to S phases (*P*<0.05, Figure 2C).

To examine the effect of ZFAS1 on cell mobility, cell migration, and invasion, assays were conducted through transwell apparatus. The results of cell migration assay showed the migrated cells in both si-ZFAS1-T24 and si-ZFAS1-RT24 groups was significantly less than the si-control-T24 and si-control-RT4 groups (*P*<0.001, Figure 2D). Consistent with cell migration assays mentioned above, the invasion assay suggested that si-ZFAS1-T24 and si-ZFAS1-RT24 groups had reduced invasive ability compared with the si-control-T24 and si-control-RT4 groups (*P*<0.001, Figure 2E).

### The potential molecular mechanism of ZFAS1 in bladder cancer

**Figure 3:**
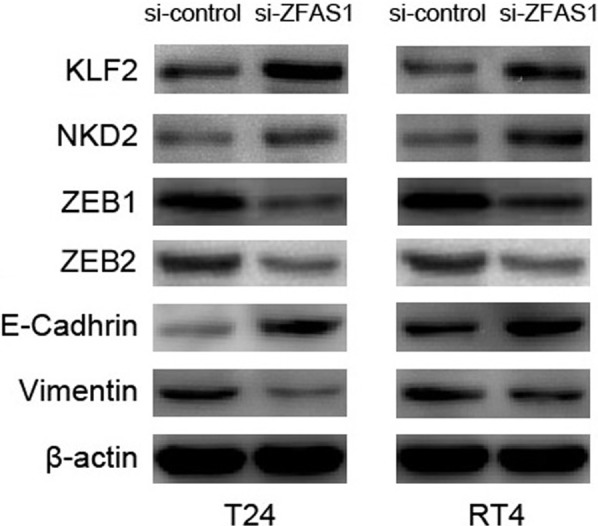
The potential molecular mechanism of ZFAS1 in bladder cancer Knocking down ZFAS1 dramatically decreases ZEB1, ZEB2, and Vimentin expressions, and increases KLF2, NKD2, and E-Cadherin expressions in T24 and RT4 cells.

Published studies indicated that ZFAS1 directly repressed KLF2 and NKD2 expression to promote tumor cell proliferation, and induced ZEB1 to enhance the epithelial–mesenchymal transition (EMT) and metastatic ability [15–17]. In order to explore the molecular mechanism of ZFAS1 in bladder cancer, we detected the effect of ZFAS1 on the expression of KLF2, NKD2, and EMT-associated genes (ZEB1, E-Cadherin, and Vimentin) by Western blot. We found that reduced ZFAS1 markedly decreased ZEB1, ZEB2, and Vimentin expressions, and increased KLF2, NKD2, and E-Cadherin expressions (Figure 3).

## Discussion

ZFAS1 is located on the antisense strand of the ZNFX1 promoter region. Originally, ZFAS1 was first identified by Askarian-Amiri et al. (in 2011) [11], at that time, it was suggested to be lowly expressed in breast cancer, and had an antitumor effect on cell proliferation and differentiation. Subsequently, Fan et al. [18] also showed ZFAS1 expression was down-regulated in breast cancer cell lines, and acted as a tumor suppressor in breast cancer. However, ZFAS1 has been suggested to be overexpressed in most types of human cancers such as lung cancer [19], hepatocellular carcinoma [20], osteosarcoma [21], glioma [22,23], colorectal cancer [24–26], gastric cancer [27–29], esophageal squamous cell carcinoma [30], ovarian cancer [31], prostate cancer [32], and acute myeloid leukemia [33]. However, the expression status of ZFAS1 in bladder cancer was still unknown. Firstly, we analyzed TCGA database, and found ZFAS1 expression was increased in bladder cancer tissues compared with paired adjacent normal tissues. Then, we confirmed ZFAS1 expression in bladder cancer tissue, normal bladder tissue, human bladder cancer cell line, and normal uroepithelium cell line, and found levels of ZFAS1 expression were elevated in bladder cancer tissues and cell lines compared with normal bladder tissues and normal uroepithelium cell line, respectively.

To assess the clinical significance of ZFAS1 in bladder cancer patients, we analyzed the correlation between ZFAS1 expression and clinical parameters in 102 bladder cancer cases, and found the expression level of ZFAS1 was positively associated with clinical stage, muscularis invasion, lymph node metastasis, and distant metastasis. Similarly, Tian et al. [19] suggested ZFAS1 expression was positively associated with TNM stage, lymph node status, and differentiation in non-small-cell lung cancer patients. In glioma patients, ZFAS1 overexpression was obviously correlated with advanced tumor grade [22]. Moreover, Fang et al. [17], Xie et al. [24], and Wang and Xing [25] consistently reported high-expression ZFAS1 was strikingly correlated with advanced clinical stage, lymph node metastasis, and vascular invasion in colorectal cancer patients. In gastric cancer, Nie et al. [15] revealed that ZFAS1 high-expression in tumor tissue was correlated with large tumor size and advanced pathological stage. Furthermore, Zhou et al. [27] and Pan et al. [28] similarly showed that circulating/serum ZFAS1 levels were significantly associated with clinical stage (TNM stage), lymph node metastasis (N classification), and distant metastasis. In addition, the analogous clinical significance of ZFAS1 was also reported in esophageal squamous cell carcinoma [30] and ovarian cancer [31].

Several meta-analyses were conducted to assess the prognostic value of ZFAS1 in human cancers, and suggested that ZFAS1 high expression might act as a credible factor for unfavorable clinical outcome in various human malignancies [34,35]. In recent years, ZFAS1 high expression has been proven as an unfavorable prognostic biomarker for many types of cancers including lung cancer [19], hepatocellular carcinoma [20], osteosarcoma [21], glioma [22,23], colorectal cancer [24,25], gastric cancer [15], esophageal squamous cell carcinoma [30], and ovarian cancer [31]. There was no study about the prognostic value of ZFAS1 in bladder cancer. In our study, a preliminarily analysis between ZFAS1 expression and overall survival showed there was no correlation (unpublished data). Moreover, we explored the prognostic value of ZFAS1 in bladder cancer patients through analyzing TCGA database, and found ZFAS1 expression had no effect on the survival time of bladder cancer patients. In order to confirm the prognostic value of ZFAS1 in bladder cancer patients, we still were collecting survival data. Besides, Chen et al. [32] showed that there was no statistical difference of survival time between high expression of ZFAS1 and low expression of ZFAS1 in prostate cancer cases.

ZFAS1 has been suggested to function as a tumor suppressor or promoter in regulating tumor cell proliferation, differentiation, apoptosis, and migration. In breast cancer, ZFAS1 overexpression obviously inhibited cell growth by inducing cell cycle arrest and apoptosis, and suppressed cell migration and invasion by repressing EMT process [11,18]. However, there is more evidence suggesting that ZFAS1 acts as an oncogenic lncRNA. Li et al. [20] demonstrated that up-regulation of ZFAS1 promoted cell invasion and tumor metastasis *in vitro and in vivo*. In osteosarcoma cell, ZFAS1 suppression markedly inhibited cell proliferation, led cycle arrest at G_0_/G_1_ phase, and promoted apoptosis *in vitro*, and inhibited the tumor growth *in vivo* [21]. In bladder cancer, ZFAS1 has been shown to be one of the targets for silibinin to regulate cell proliferation, migration, invasion, and apoptosis [36]. However, the biological role of ZFAS1 in bladder cancer cell was unknown. In our study, loss-of-function studies indicated ZFAS1 suppression obviously decreased cell proliferation, migration and invasion, and reduced cell cycle progression from G1 to S phases. Furthermore, Nie et al. [15] reported that NKD2 and KLF2 were key downstream mediators of ZFAS1 in modulating tumor cell proliferation. Liu et al. [16] and Fang et al. [17] suggested ZFAS1 modulated ZEB1 and ZEB2 expression to regulate tumor cell migration, invasion, and EMT process. In order to explore the molecular mechanism of ZFAS1 in regulating bladder cancer cell proliferation, migration, and invasion, we detected the effect of ZFAS1 on the expression of KLF2, NKD2, and EMT-associated genes (ZEB1, ZEB2, E-Cadherin, and Vimentin), and found that reduced ZFAS1 markedly decreased ZEB1, ZEB2, and Vimentin expressions, and increased KLF2, NKD2, and E-Cadherin expressions. Thus, knockdown of ZFAS1 repressed bladder cancer cell proliferation via up-regulating KLF2 and NKD2 expression, and inhibited cell migration and invasion via down-regulating ZEB1 and ZEB2 expression.

In conclusion, ZFAS1 overexpression is observed in bladder cancer tissues and cell lines, and associated with aggressive progression in bladder cancer patients. Knockdown of ZFAS1 expression inhibits cell proliferation migration and invasion via regulating KLF2, NKD2, ZEB1, and ZEB2 expression.

## Abbreviations

EMT: epithelial–mesenchymal transition
lncRNA: long non-coding RNA
TCGA: the Cancer Genome Atlas
ZFAS1: zinc finger antisense 1
ZNFX1: zinc finger NFX1-type containing 1
